# Neuromechanical interactions between the limbs during human locomotion: an evolutionary perspective with translation to rehabilitation

**DOI:** 10.1007/s00221-016-4715-4

**Published:** 2016-07-15

**Authors:** E.P. Zehr, Trevor S. Barss, Katie Dragert, Alain Frigon, Erin V. Vasudevan, Carlos Haridas, Sandra Hundza, Chelsea Kaupp, Taryn Klarner, Marc Klimstra, Tomoyoshi Komiyama, Pamela M. Loadman, Rinaldo A. Mezzarane, Tsuyoshi Nakajima, Gregory E.P. Pearcey, Yao Sun

**Affiliations:** 1 Rehabilitation Neuroscience Laboratory, University of Victoria, PO Box 3010 STN CSC, Victoria, BC Canada V8W 3P1; 2 Human Discovery Science, International Collaboration on Repair Discoveries (ICORD), Vancouver, BC Canada; 3 Centre for Biomedical Research, University of Victoria, Victoria, BC Canada; 4 Division of Medical Sciences, University of Victoria, Victoria, BC Canada; 5 Department of Pharmacology-physiology, Faculty of Medicine and Health Sciences, University of Sherbrooke, Sherbrooke, QC Canada; 6 Department of Physical Therapy, SUNY Stony Brook University, Stony Brook, NY USA; 7 Division of Sports and Health Science, Chiba University, Chiba, Japan; 8 The United Graduate School of Education, Tokyo Gakugei University, Tokyo, Japan; 9 Department of Integrative Physiology, Kyorin University School of Medicine, Tokyo, Japan; 10 Motion and Mobility Rehabilitation Laboratory, University of Victoria, Victoria, BC Canada; 11 Laboratory of Signal Processing and Motor Control, College of Physical Education, Universidade de Brasília—UnB, Brasília, Brazil

**Keywords:** Evolution, Rehabilitation, Neuroscience, Walking, Primate, Reflex, CNS

## Abstract

During bipedal locomotor activities, humans use elements of quadrupedal neuronal limb control. Evolutionary constraints can help inform the historical ancestry for preservation of these core control elements support transfer of the huge body of quadrupedal non-human animal literature to human rehabilitation. In particular, this has translational applications for neurological rehabilitation after neurotrauma where interlimb coordination is lost or compromised. The present state of the field supports including arm activity in addition to leg activity as a component of gait retraining after neurotrauma.

…all parts of the nervous system are connected together and no part of it is probably ever capable of reaction without affecting and being affected by various other parts….

– Sir Charles S. Sherrington, “Integrative Action of the Nervous System” (1906) (Sherrington 1906).

## Introduction

Smooth, coordinated movements between the limbs are motor outputs that most people take completely for granted. Despite the sublime interactions between the limbs, the nature of interlimb locomotor coordination has been an area of uncertainty in motor control research. Certainly, both voluntary and involuntary mechanisms are involved in this finely tuned coordination. Sir Charles Sherrington articulated the view that subconscious mechanisms support the generation and control of many kinds of locomotor movement within the “integrative action of the nervous system” (Sherrington 1906).

During human walking, one leg must support the body in stance, while the other limb moves through the swing phase to facilitate forward progression. The critical role of this integration is clearly revealed by its dysregulation in neurotrauma and resulting uncoordinated and inefficient locomotion. When normal walking coordination is disrupted by a stroke or other injury, walking function often suffers. Indeed, the degree of asymmetry in walking coordination following stroke correlates with stages of motor recovery, walking speed and number of falls (Brandstater et al. 1983; Olney et al. 1994; Titianova and Tarkka 1995). The purpose of this review is to summarize evidence from cats and non-human related to interlimb coordination and extend this to arm and leg interactions during human locomotion. This is centred around concepts related to evolutionary conservation of mechanisms, functions of arm and leg coupling during locomotion, and translation of this work to clinical neurorehabilitation. Regaining locomotor function after stroke, spinal cord injury (SCI), and traumatic brain injury, amongst others, is a primary goal in rehabilitation. Understanding and exploring preserved evolutionary interlimb connections may provide an opportunity to optimize functional recovery and quality of life.

## Arm and leg coupling in an evolutionary context

The most frequent accompanying animal-like manifestation among the children who develop running on all fours, is climbing.

Ales Hrdlicka in “Children Who Run On All Fours” (Hrdlicka 1931)

On the African savanna, standing up on the hindlimbs and walking upright in bipedal locomotion may have emerged as a survival advantage in viewing, intercepting or avoiding predators, prey, and foraging items [see summary in (Sockol et al. 2007)]. For example, bipedal locomotor behaviour in the chimpanzee and orangutan may have arisen from arboreal foraging (Thorpe et al. 2007). When seeking fruit while climbing horizontally on branches, these primates often stand up and grasp at branches to enhance stability. The more slender distal branches offer less support reinforcing the need to seek stability by grasping a branch with the upper limbs. The coupling between the limbs had to change from locomotor control to parallel, symbiotic systems used for posture, support and gathering. This contributor to the acquisition of bipedal locomotor behaviour in primates parallels development in human infants when upright standing and locomotion are acquired (Ivanenko et al. 2007), as both behaviours are related to exploration of novel environments. Environmental cues may influence both human developmental acquisition of bipedalism as well as the exploitation of bipedal locomotion on an evolutionary timeline.

In physical anthropology, it is accepted that the divergence between hominids and our closest cousins, the chimpanzees, occurred about 5–7 million years ago. Using the famous Laetoli footprints near Olduvai Gorge in Tanzania as a fossil record benchmark, it appears skilful bipedal locomotion has been present for at least the past ~3.7 million years. These footprints are important as there are no concomitant knuckle-walking impressions suggesting some skill in bipedal walking. Additional study of fossil records suggests onset of regular bipedal activity as early as 7–9 million years ago (Kohler and Moya-Sola 1997). Regardless of the exact timeline, we have clearly been using upright bipedal locomotion throughout human history. Nonetheless, a simple glance at any playground where climbing apparatuses (including “monkey bars”) are found reveals that we humans have retained a strong desire to climb. Niemitz suggests “It seems to be a trait of human behavioral genetics that *Homo sapiens* goes through an ontogenetic stage when climbing is an important and much liked part of the locomotor repertoire” (Niemitz 2002). The adaptive value of climbing or arboreal walking in humans may seem abstract in the modern urban environment. However, the ubiquitous nature of climbing and arboreal walking behaviours across primates provides context for conserving coordinated linkages between the arms and legs in humans (Zehr et al. 2009). What do we now know about the implications of evolution on the neural coordination of arm and leg movements in modern day humans?

### A relationship between human bipedal interlimb coupling and quadrupedal locomotor coordination?

To go on two legs is very hard. Perhaps four is better, anyway

Sayer of the Law in “The Island of Dr. Moreau” (1996)

**Fig. 1:**
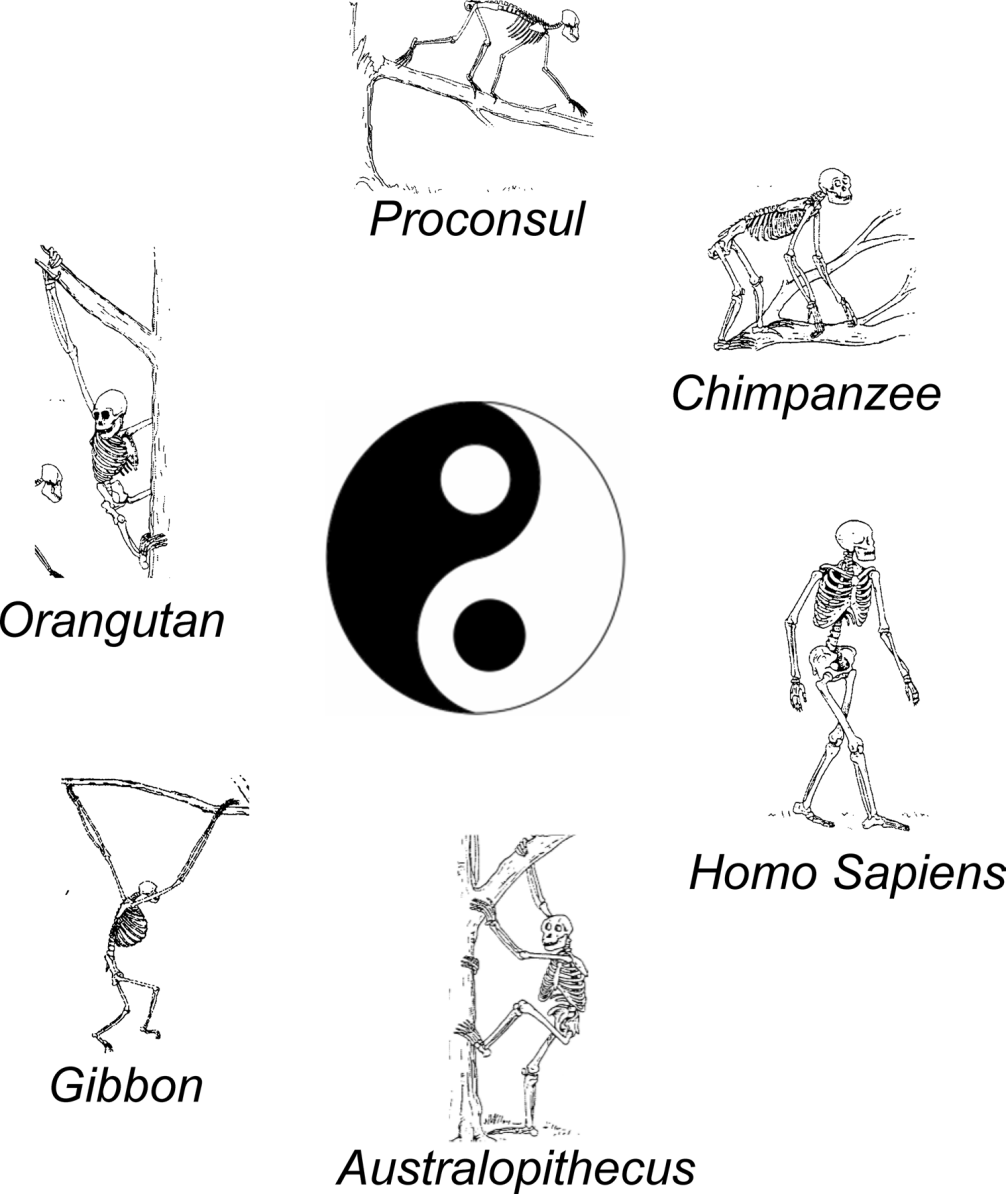
Some primate quadrupedal and bipedal locomotor behaviours

Some obvious biomechanical and functional differences exist between bipedal and quadrupedal gait (e.g. see Fig. 1 for examples of the primate locomotor repertoire). In bipedal locomotion, the centre of mass is relatively high and balanced on only two legs making the role of each leg critical. Also, bipedal gait can be performed in the absence of propulsive activity of the arms, thus freeing the arms to perform independent, skilled hand movements during locomotion. Despite these differences, the persistent preservation of the neuronal connections between rhythmic arm and leg movement may be related to the biomechanics of upright locomotion.

Studying the bipedal locomotion of non-human primates is important for clarifying the evolution of habitual bipedalism in the human lineage. An intriguing capability of Macaca Fuscata (Japanese monkey) is that it can transform its locomotor patterns from quadrupedal to bipedal and vice versa without a break in forward speed on a moving treadmill belt (Nakajima et al. 2004). Mori et al. (2001) also found that when these monkeys are trained to walk bipedally and have to clear an obstacle, the kinematics of the trailing limb change according to the obstacle’s height and position relative to the leading limb. When the monkey stumbled over an obstacle, it used a “defensive posture” (which includes a rapid lowering of the foot and simultaneous forelimb movement) to stabilize the perturbed posture and prevent falling. The monkey then raises its centre of mass to the appropriate level for continuation of normal bipedal walking. These results indicate that *M. fuscata* recruits both anticipatory and reactive neural mechanisms to accommodate the obstacle by rapidly selecting and combining integrated subsets of posture and locomotor-related networks appropriate for the task at hand (Mori et al. 2001). In the presence of electrically simulated (Haridas and Zehr 2003) obstacles or mechanical impediments to stepping, humans similarly demonstrate anticipatory alterations as assessed by cutaneous reflexes evoked from the distal tibial nerve in proximal arm muscles, suggesting a quadrupedal coordination during skilled locomotion (Michel et al. 2008).

In order to achieve the conversion from quadrupedal to bipedal locomotion, *M. fuscata* utilizes a new hip joint motion, which generates propulsive force in a fashion quite similar to that of the human (Nakajima et al. 2004). Kinematic analyses of *M. fuscata*’s bipedal locomotion confirmed this animal has similar kinematics to humans for the integration of posture and locomotion, particularly for the hip joint (Mori et al. 2004a, b). Nakajima et al. (2004) established that the basic relationships between the stance and swing phases of the step cycle frequency are essentially the same for quadrupedal and bipedal walking. They interpreted this finding as evidence that *M. fuscata* employs quadrupedal locomotor networks for the elaboration of bipedal locomotion. In addition, higher central nervous system (CNS) mechanisms may be utilized for the selection of an appropriate postural strategy and limb-kinematic parameters to move the weight-bearing hindlimbs (Nakajima et al. 2004).

Ogihara et al. (2007) tracked ground-reaction-force (GRF) profiles of bipedally trained Japanese macaques (performing monkeys) during bipedal locomotion. Trained monkeys exhibited vertical GRF profiles with a single peak that were similar to those of ordinary monkeys. They did not generate the double peaked (heel contact and toe off) force curve that is seen in humans, despite their extensive training. However, in the trained monkeys, the peak occurred earlier in the stance phase, and the overall shape was more triangular than that of the more parabolic profile generated by ordinary monkeys. Further kinematic analysis compared highly trained and ordinary macaques during bipedal locomotion. The trained macaques walked with longer and less frequent strides and appeared to use inverted pendulum mechanics during bipedal walking, which resulted in an efficient exchange of potential and kinetic energy.

Recent findings may provide insight into the early stages of human bipedalism. Nakatsukasa (2004; Nakatsukasa et al. 2004, 2006) showed that the metabolic cost of bipedal locomotion in bipedally trained monkeys is approximately 20–30 % higher than that of quadrupedal locomotion (i.e. their inherent mode of locomotion). However, if functional benefit from increased energy return acquired by habitual bipedal locomotion outweighs the energetic cost, such an advantage could be a factor applying selective pressure for bipedal locomotion. A recent study by Ogihara et al. (2011) explored dynamically reconstructed bipedal walking of Japanese macaques by computer simulation to investigate causal relationships among kinematics, kinetics and energetics of bipedal locomotion. They found the metabolic cost of locomotion decreased as speed increased during bipedal walking, and the cost of transport was reduced when vertical displacement of the hip joint was virtually modified to be more like the upright bipedal human. This suggests that human vertical fluctuations in the body’s centre of mass actually contribute to energy savings via an inverted pendulum mechanism. However, the shift to truly humanlike locomotion with full utilization of the inverted pendulum mechanism would require the evolution of an appropriate hip morphology. The anatomically restricted range of hip joint motion in quadrupedal monkeys impedes the generation of humanlike bipedal locomotion. This indicates morphological rearrangement of the hip joint is an essential precondition for protohominids to acquire humanlike bipedalism (Ogihara et al. 2007).

To assess the natural ability of a quadrupedal primate’s ability to walk bipedally, Ogihara et al. (2005) made kinematic comparisons between *M. fuscata* monkeys, which were highly trained to walk upright, and a monkey, which spontaneously acquired bipedal locomotion after loss of forearms and hands due to a congenital malformation. Results showed that all joints were relatively more flexed in the untrained monkey. It was also noted that the ankle was less plantar flexed and the knee more flexed in the mid-to-late stance phase in the untrained monkey, suggesting that the trunk was not lifted up to store potential energy. In the trained monkeys, the joints were extended to bring the trunk as high as possible in the stance phase, and then, stored potential energy was exchanged for kinetic energy to move forward. The efficient inverted pendulum mechanism seemed to be absent in the untrained monkey’s locomotion, implying that acquisition of such efficient bipedal locomotion is not a spontaneous ability for a Japanese monkey. This appears to be a special skill that can only be acquired through artificial training for an inherently quadrupedal primate (Ogihara et al. 2005). Further data on neuronal mechanisms underlying the acquisition of bipedal locomotor control in habitual quadrupeds are anticipated. Recently, Hosoido and colleagues evaluated the effect of bipedal walking training on interlimb reflex modulation in the rat (Hosoido et al. 2011). Excitability of H-reflexes in forelimbs and hindlimbs was modulated as a function of training, an outcome suggested to arise as function of interlimb neuroplastic adaptation due to bipedal training.

### Emergence of bipedal locomotor control

It is possible that the reciprocal coordination of the arms for use in quadrupedal climbing evolved into use of the arms for offsetting interaction torques generated during upright bipedal terrestrial locomotion. Oxnard and Franklin took an approach based on muscle morphology to consider changes in head, neck and forearm musculature associated with transition from quadrupedal locomotion in non-human primates to upright bipedal locomotion in modern humans (Oxnard and Franklin 2008a, b). Based upon their detailed anatomical comparisons and the identification of myosin gene mutations in masticatory muscles in non-human and human primates observed by Stedman and colleagues (Stedman et al. 2004), it seems reasonable that the morphological correlates of habitual upright locomotion emerged approximately 2.4 million years ago. A remaining functional implication of this transition from quadrupedal to bipedal locomotion is seen in the work of Hinrichs and colleagues (Hinrichs 1987; Hinrichs et al. 1987). They suggest that significant whole-body angular momentum around a vertical axis can be induced by the lower body motion during human walking/running. This is offset by active upper body motion to minimize perturbations and suggested to require neuronal coordination (Zehr and Duysens 2004).

One of the first cases to document human quadrupedal locomotion was documented by the photographer Eadweard Muybridge where he highlighted a child walking on all four limbs with a paralysed leg as a result of polio (Muybridge 1887). In healthy adult humans, quadrupedal locomotion is characterized by interindividual variability.

More recently, a syndrome has been detailed (Tan 2007, 2010), which includes four main characteristics including diagonal-sequence quadrupedal gait, impaired intelligence, rudimentary language skills and primitive conscious experience. Diagonal-sequence quadrupedal locomotion is highlighted by a foot touching the ground followed by the contralateral hand and vice versa. Individuals with this syndrome are able to stand and remain upright as long as they do not try to move. However, initiating the necessary movement to take a step produces an immediate loss of balance due to disturbances in cerebellar function (Tan 2010). An early suggestion is that in these affected individuals, activation of ancestral quadrupedal locomotor networks or a return to an infantile development stage allows for an adaptive response, which has been preserved for approximately the last 400 million years (Shapiro and Raichlen 2005; Karaca et al. 2012). However, based on gait analysis of 518 quadrupedal strides of individuals with this syndrome, it has alternatively been suggested that they only use lateral-sequence gait, which is similar to that of healthy human adults (Shapiro et al. 2014). Thus, this evidence supports that the quadrupedal gait observed in persons with this syndrome can be best explained by biomechanical principles rather than “devolution”.

### Coordination between the legs

While human bipedal locomotor networks evolved over time, many preserved quadrupedal features remain intact and may be accessible under the right set of environmental circumstances. Interlimb coordination is an inherent property of human neural circuitry. A substantial body of literature has identified that muscle activity and force production in a particular muscle can be modified by movement in the opposite limb, as evidenced by studies of reflex modulation during rhythmic movement. For example, both active and passive movements of the contralateral leg substantially inhibit the ipsilateral soleus H-reflex (Brooke et al. 1992; Collins et al. 1993; Cheng et al. 1998; Misiaszek et al. 1998). Also, during walking, running and leg cycling, cutaneous reflexes are modulated in both the contralateral and ipsilateral limbs (Duysens et al. 1992; Brown and Kukulka 1993; Tax et al. 1995). Ting et al. observed that the muscle activity and force generated by a leg during unilateral cycling was dependent on the activity and sensory state of the other limb. This was demonstrated even though the forces experienced during unilateral cycling were matched to bilateral cycling conditions (Ting et al. 1998a, b).

Experiments using split-belt treadmills in adults have shown that the legs maintain alternating coordination with belts running at different speeds (Dietz 1994; Prokop et al. 1995; Erni and Dietz 2001). These studies confirm a strong bilateral coupling of the legs necessary for efficient and stable locomotion. The regulation of walking requires a close bilateral coordination of muscle activation in the legs. Indeed, the coordination between the legs during gait may be more critical for humans than for other animals given the added challenge of stability in bipedal walking. It has been known for some time that there is interdependence between the pattern generators controlling the movement of each side in animals. Grillner and Rossignol (1978) found that the initiation of the swing phase was dependent on the position of the contralateral leg: swing could only be initiated in spinalized cats when the other leg was either in mid-swing or mid-stance (Grillner and Rossignol 1978).

Additionally, disturbances that prolong the swing phase in cats have been shown to cause compensatory reactions in the contralateral limb in order to preserve ground contact and stability (Forssberg et al. 1977; Matsukawa et al. 1982). Also in the cat, the response to stepping into a hole was different depending on the state of the contralateral leg: if the contralateral leg was in stance, this stance phase was extended until the ipsilateral side regained ground contact; if the contralateral leg was in swing, the corrective flexion response on the ipsilateral side was delayed until the contralateral side regained ground contact (Gorassini et al. 1994; Hiebert et al. 1994).

Interlimb coordination between the two legs in humans bears some resemblance to that in other animals. For example, when the swing phase is initiated early in one limb (via a rapid extension of the hip), swing on the contralateral side is truncated so that this limb can regain ground contact (Berger et al. 1984; Dietz et al. 1984; Pang and Yang 2001). Coordinated responses in both legs are generated in response to a stumble or perturbation during swing in both adults and infants (Dietz et al. 1986; Eng et al. 1994; Yang et al. 1998a, b; Schillings et al. 2000).

**Fig. 2:**
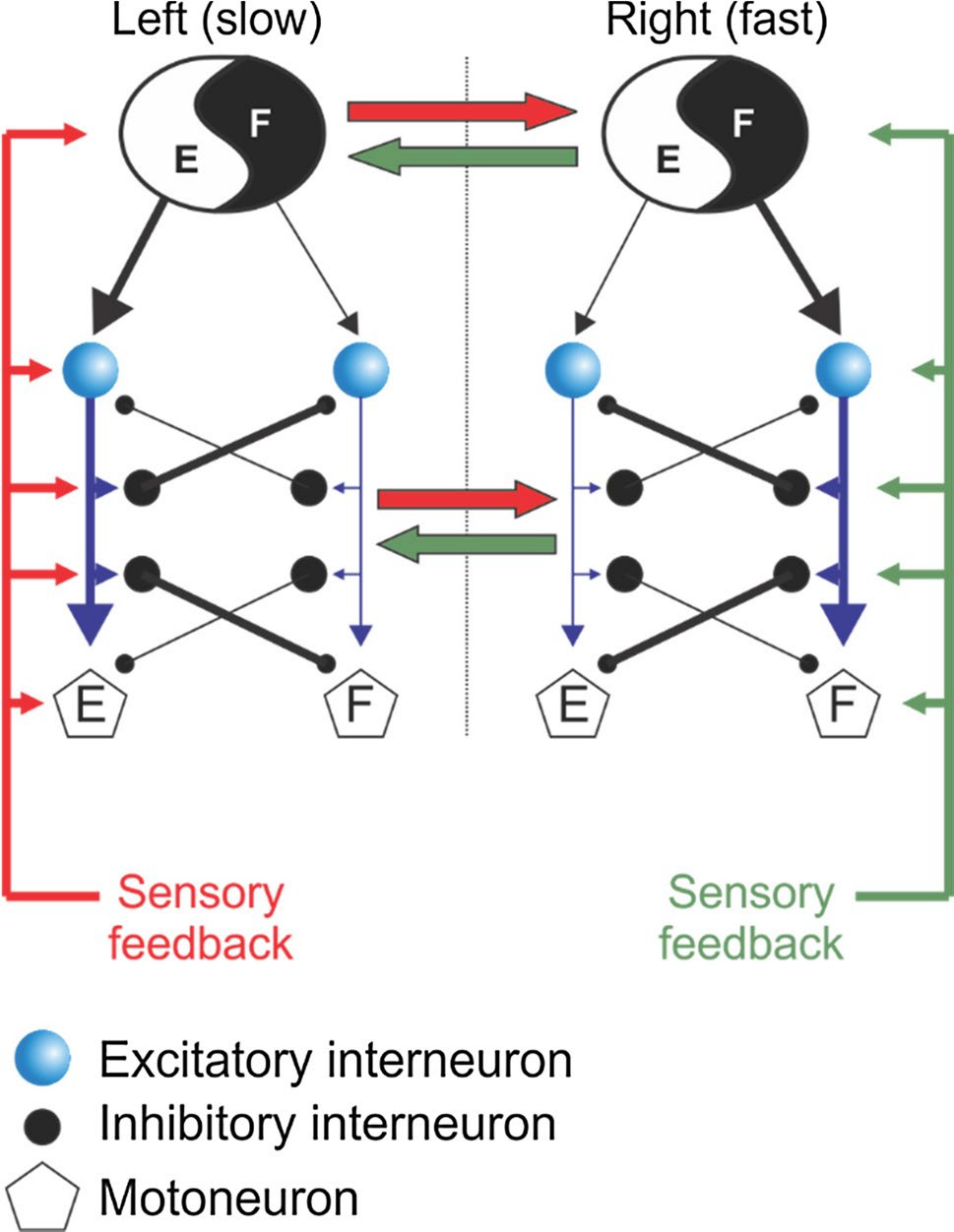
Idealized schematic of neuronal interactions subserving interlimb coordination during locomotion

There is a similar interaction between the two legs to maintain 1:1 (i.e. equal number of steps on left and right sides) alternating stepping on a split-belt treadmill in human infants and adults (Thelen et al. 1987; Dietz et al. 1994b; Prokop et al. 1995; Yang et al. 2005). Stance duration of the leg stepping on the slower belt is longer than that of the leg stepping on the faster belt. The longer slow-belt stance duration is coupled with a longer swing duration on the contralateral (fast belt) side. In addition to humans, this relationship has been shown in intact (Halbertsma 1983; D’Angelo et al. 2014), decerebrate (Kulagin and Shik 1970) and spinal-transected (Forssberg et al. 1980; Frigon et al. 2013) cats. Thus, the ability to couple stance and swing phases bilaterally can be accomplished at a spinal level, most likely via asymmetric sensory feedback from peripheral mechanoreceptors. For instance, in the neurologically intact cat, limb extensor activity is greater on the slow side, indicating greater feedback from load-related inputs while the fast moving limb would undergo higher rates of stretching and hence greater feedback from stretch-related inputs (Frigon et al. 2015). A schematic representation showing the influences of sensory feedback from the left and right sides onto spinal locomotor pattern generators is shown in Fig. 2.

Coupling of the legs does not always result in stepping patterns that are bilaterally symmetrical. With an increasing left–right speed differential during split-belt locomotion, the limb on the fast belt can take more steps than the limb on the slow belt in human infants (Thelen et al. 1987; Yang et al. 2005) and spinalized cats (Frigon et al. 2013) Furthermore, bilateral coupling of the legs is not limited to rhythmic output that would result in forward progression. Human adults (Choi and Bastian 2007) and infants (Yang et al. 2005) can perform forward stepping with one limb and backward walking with the other. Although these results would suggest that the left and right spinal pattern generators can operate autonomously, it should be noted that swing phases never overlap, and the phasing between contact of the left and right limbs remains very consistent, even during 1:2, 1:3, 1:4 and 1:5 left–right step relationships. This indicates tight coupling between the left and right locomotor pattern generators, even under extreme circumstances. This tight coupling seems vital to the maintenance of balance and progression during locomotion.

### Coordination between the arms

In habitual quadrupeds, forelimb movements are thought to be coordinated by central pattern generators (CPGs) in the cervical spinal cord that are similar to those in the lumbar spinal cord coordinating hindlimb movement (Yamaguchi 2004; Zehr et al. 2004). Substantial evidence has accumulated, supporting that rhythmic arm movements in humans are also coordinated in part by spinal networks that are similar to those controlling rhythmic leg movements (Zehr and Duysens 2004). For example, cutaneous reflexes evoked in arm muscles during arm swing of walking and rhythmic arm cycling show phase modulation that is generally unrelated to background muscle activity and, thus, indicative of premotoneuronal gating by spinal networks (Zehr and Kido 2001; Zehr and Haridas 2003).

While not as apparent in the bipedal human, quadrupedal interlimb coupling can be observed in the natural movement of the arms (arm swing) that occurs during gait. In an early investigation of arm swing during walking, Elftman (1939) calculated the muscle torque of the arms during gait. Elftman’s conclusion that arm swing was not simply a passive pendular action, but required active muscle contractions were later corroborated by the suggestion that active motor output in the arms was a component of arm swing during walking (Fernandez-Ballesteros et al. 1965; Hogue 1969). Arm muscles were rhythmically active even while mechanically restrained during walking (Fernandez-Ballesteros et al. 1965; Kuhtz-Buschbeck and Jing 2012).

Recent work has shown that arm swing is highly correlated with increase trunk torsion, whereas it is not inversely correlated with pelvis torsion during running (Pontzer et al. 2009). Furthermore, EMG activity of the anterior and posterior deltoids plays a role in dampening excessive arm swing during running. These results suggest that passive arm swing may predominate over active arm swing during running, but these results were not as clear during walking. Therefore, arm swing during human walking likely arises through an active component interacting with the passive arm motion generated by interaction torques as a result of leg movement. Recent work exploring the mechanisms and functions of arm swing in human gait concluded that arm swing should be seen as an integral part of human bipedal locomotion (Meyns et al. 2013; Massaad et al. 2014). Arm swing arises mostly from passive movements and is stabilized by active muscle control originating from locomotor circuits in the central nervous system. The central nervous system must account for active and passive components to control the limbs and produce smooth locomotion (Jackson 1983; Jackson et al. 1983a, b). Evidence from reflex studies again supports the existence of active neuronal regulation of arm swing during locomotion (Zehr and Haridas 2003).

While there are similarities between the neural control of rhythmic upper and lower limb movements in humans, the degree to which the left and right sides are coupled is different between the arms and legs: the coupling between the arms is weaker than between the legs. For instance, while passive movement of the contralateral leg suppressed H-reflexes in the stationary leg (Collins et al. 1993; Cheng et al. 1998; Misiaszek et al. 1998), there was little effect of contralateral arm movement on H-reflex amplitude in the stationary arm (Zehr et al. 2003).

Comparable studies of arm-to-arm coupling conducted during rhythmic arm movement have revealed weaker bilateral coordination compared to the legs, consistent with their minor role in postural stability and forward progression in humans (Zehr and Duysens 2004; Carroll et al. 2005; Vasudevan and Zehr 2011). Cutaneous reflex modulation in the arms during cycling movements is relatively unaffected by the activity of the contralateral arm (Carroll et al. 2005), and crossed effects emerge only with extreme timing differences [asynchronous coupling 2 Hz:1 Hz; (Vasudevan and Zehr 2011)]. This suggested that while there was evidence of a similar central regulation of rhythmic arm and leg movement, the coupling between the arms was weaker than that noticed in the legs. This conclusion is supported by earlier observations where active contralateral arm movement inhibited the ipsilateral forearm H-reflex in a stationary limb while passive movement of the contralateral limb did not (Zehr et al. 2003). This evidence suggests reduced involvement of afferent feedback on the weak contralateral reflex suppression.

Cutaneous reflex modulation patterns during arm cycling were found to be highly conserved regardless of differences in EMG activity (Carroll et al. 2005), movement symmetry (Hundza and Zehr 2006) and asynchronous coupling on the contralateral side (Vasudevan and Zehr 2011). Therefore, cutaneous and H-reflexes in the arms seemed primarily dependent on the state of the limb in which the muscle resides, whereas reflexes in the legs were also affected by the functional state of the contralateral limb.

Functionally, it is perhaps not surprising that there is stronger coupling between the two legs. During bipedal walking, the legs perform symmetric, coordinated, out-of-phase locomotor movements while the arms have the option of moving independently of one another. The roles of the arms and legs in human walking are different, and the strength of coupling between pattern generators controlling each limb may have developed to reflect this.

### Coordination between the arms and legs

In quadrupeds, there is a bidirectional coupling between lumbar and cervical spinal locomotor centres (Gernandt and Megirian 1961; Gernandt and Shimuamura 1961; Skinner et al. 1980; Ballion et al. 2001; Juvin et al. 2005; Thibaudier et al. 2013; Thibaudier and Frigon 2014). For instance, during transverse split-belt locomotion with the forelimbs and hindlimbs stepping at different speeds from one another in intact cats, adjustments in the pattern are strikingly different depending on the girdle that is stepping faster (Thibaudier et al. 2013; Thibaudier and Frigon 2014). When the forelimbs step faster, they start taking more steps than the hindlimbs (2:1 fore–hind step relationship), even with small fore–hind speed differentials. Despite the appearance of 2:1 fore–hind step relationships, consistent phasing between homolateral and diagonal limbs is maintained, indicating tight coupling between cervical and lumbar pattern generators. When the hindlimbs step faster, even with large fore–hind speed differentials, a 1:1 fore–hind step relationship is always maintained. The hindlimbs adjust to faster speeds on the transverse split-belt by increasing stride length, while the forelimbs adopt a faster cadence. This undoubtedly reflects mechanical constraints imposed on cervical and lumbar spinal neural circuits, as the hindlimbs can travel a longer distance backwards to make adjustments to speed compared to the forelimbs.

The neural mechanisms involved in cervicolumbar communication during locomotion remain largely unknown in mammals. They most likely involve long and short pathways intrinsic to the spinal cord (propriospinal) that are activated by supraspinal, spinal and sensory inputs. In fact, the motor cortex has been identified to be involved in monitoring the interlimb coordination of forelimb and hindlimbs during locomotion of cats (Zelenin et al. 2011). The role of the propriospinal system in intergirdle coordination was investigated in the isolated spinal cord of neonatal rats during fictive locomotion (Juvin et al. 2005, 2012). In vitro recordings from cervical and lumbar ventral roots showed coordinated activities. It is important to note the absence of signals from supraspinal structures and movement-related feedback in these preparations. When a synaptic blockade was applied to thoracic segments, cervical and lumbar rhythms became independent, although they remained stable and continued to discharge at similar frequencies (Juvin et al. 2005). This reduction in synaptic transmission within mid-thoracic regions did not affect the transmission of long propriospinal pathways that passed through, indicating that these pathways are not sufficient by themselves for intergirdle coordination, at least in neonatal rats. Instead, it was proposed that intergirdle coordination in neonatal rats is mediated by short-projecting propriospinal neurons (Juvin et al. 2012).

During human locomotion, Donker et al. observed changes in limb coordination after adding mass to the wrist or ankle. Adding a mass to the wrist during walking resulted in increased muscle activity in both arms and a decrease in the movement amplitude in only the loaded limb. Adding load to an ankle, however, produced increased muscle activity and movement amplitude in both arms (Donker and Beek 2002; Donker et al. 2002).

Using a combined arm and leg cycling task with mechanically independent ergometers, arm cycling cadence was modified by changes in leg cycling cadence. However, changes in arm cycling cadence did not alter leg cycling cadence suggesting an ascending directional bias in locomotor coupling (Sakamoto et al. 2007). MacLellan et al. (2013b) examined upper and lower limb coordination during lower limb asymmetry in a split-belt walking paradigm with four different speed ratios (2:2, 2:4, 2:6 and 2:8 km/h) and the left belt fixed at 2 km/h. While amplitudes of the right lower limb increased and left lower limb decreased with increasing asymmetry, both upper limb amplitudes increased. Correlations between diagonal upper/lower limb trajectories increased as right belt speed became faster, suggesting increasing cross-body matching regardless of side. As the treadmill asymmetry increased, ipsilateral limbs became more out of phase suggesting a more precise gait pattern to regulate timing between limbs. It appears the faster moving lower limb drives the motion of both upper limbs. Although these findings are currently limited to the split-belt paradigm, the changes are most likely due to neural mechanisms in which upper and lower limb CPGs regulate full-body movement and maintain the rhythmic locomotor pattern.

**Fig. 3:**
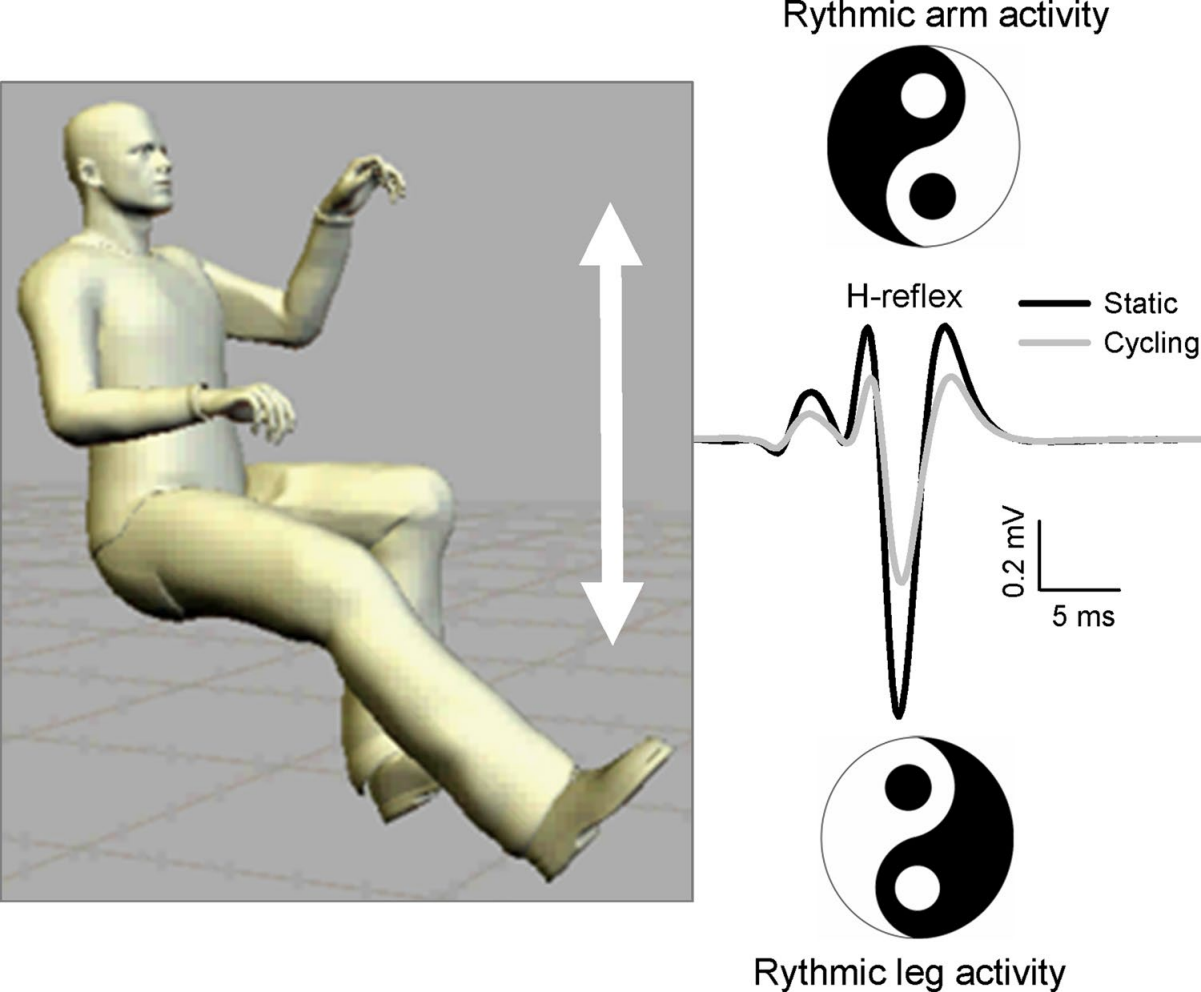
Schematic distillation of evidence showing reciprocal organization of remote effects of rhythmic arm cycling. Rhythmic arm activity modulates H-reflexes in the legs, and rhythmic leg activity modulates H-reflexes in the arms

Effects of rhythmic arm movement on motor output in the lower limb have been demonstrated in various ways in the literature, including remote influences on reflex excitability. For example, performing rhythmic walking of the hands on an overhead treadmill when humans are lying on their side induces locomotor-like activity of the legs (Sylos-Labini et al. 2014). Furthermore, numerous studies have monitored H-reflex modulation in leg muscles induced by rhythmic arm cycling (Frigon et al. 2004; Loadman and Zehr 2007; Dragert and Zehr 2009; Hundza and Zehr 2009; de Ruiter et al. 2010; Hundza et al. 2012) and reflexes in the arm during rhythmic leg cycling (Carroll et al. 2005) (see Fig. 3).

Sidhu and colleagues used transcranial magnetic stimulation to evoke motor evoked potentials during and after leg cycling. They found that excitability of lower-threshold corticospinal projections to the elbow flexor muscles was reduced with pharmacological blockade of feedback from group III/IV muscle afferents. During leg cycling (presumably providing strong activation of the group III/IV afferents from the active leg muscles), there was facilitation of corticospinal projections to upper limb muscles (Sidhu et al. 2014). It was suggested that this increase in responsiveness could be accounted for by neural coupling between arms and legs during rhythmic movements in humans.

Insight may be gained on responses in ankle flexor muscles from work that has studied effects of arm movement on lower leg cutaneous reflexes. One study employed arm/leg cycling and observed that cutaneous reflexes in many leg muscles, including TA, were significantly modulated by arm movement when combined with local leg movement (Balter and Zehr 2007). Similar modulatory effects of arm movement on cutaneous reflexes in the legs are also observed during combined arm/leg recumbent stepping (Zehr et al. 2007a).

Other evidence of arm movement modulatory effects on the lower limb has emerged from studies examining leg EMG recordings. During combined arm/leg recumbent stepping, active arm movement has been found to significantly increase activation in a number of leg muscles, including tibialis anterior (TA) and soleus (SOL) (Huang and Ferris 2004; Ferris et al. 2006). This was not seen when the legs were externally passively driven yet remained when the arms were active and mechanically uncoupled from the legs. Using the same arm and leg stepping device, Kao and Ferris found that increased movement frequency enhanced the lower limb activation induced by arm activity (Kao and Ferris 2005). These results support spinal interlimb connections mediated through propriospinal neural circuitry (Dietz 2002).

A spinal mechanism of interlimb coordination was inferred by Kawashima et al. (2008) in a study of an incomplete spinal cord injury. Arm swing significantly altered SOL activation during rhythmic leg movement, to better emulate stereotypical patterns normally seen during different walking phases. Further, these effects were observed during both passive and active conditions, leading the authors to suggest that spinal input from upper limb movement played a significant role in shaping SOL motor output. Since the studies were in people with incomplete cervical SCI, however, the impact of preserved descending drive cannot be excluded.

The intrinsic efficiency of arm and leg coordination during human locomotion has been examined metabolically. Meyns et al. (2014) examined which combination of arm and leg movements were more efficient during simultaneous arm and leg cycling. Twenty-five able-bodied adults performed eight submaximal tests of 6 min on a hybrid handcycle during four different conditions (‘arms only’ or ‘arms and legs’ with arms symmetrically in phase or asymmetrically out of phase). Oxygen uptake (VO2), heart rate (HR) and Borg score (Borg) were assessed. During ‘arms only’, no differences were found in exercise intensity between symmetrical and asymmetrical movements. In contrast, during ‘arms and leg’, both VO2 and Borg were significantly lower for the asymmetrical compared to the symmetrical condition. This study demonstrated that asymmetrical arm movements, especially in combination with leg movements, represented the most efficient condition on a stationary hybrid handcycle and suggests that neural energy costs are lower when moving in the preferred (asymmetrical) coordination.

It has been established that arm movements during human gait affect leg activity due to neural coupling between arms and legs. Based on previous work, it may be expected that patterns of locomotor-like alternating arm swing should be more effective than in-phase swing at altering motor output in the legs. Massaad et al. (2014) revealed that soleus H-reflexes were suppressed for all arm, trunk or leg movements, but a distinct and marked reflex modulation occurred during locomotor-like anti-phase arm swing. This modulation had a peculiar bell shape and showed maximum suppression at a moment where the heel strike would occur during a normal walking cycle and was independent from background electromyographic activity, suggesting a spinal processing at a premotoneuronal level. Interestingly, De Ruiter et al. (2010) also showed a bell-shaped modulation curve for soleus H-reflexes during arm cycling supporting the concept that similar neural mechanisms produce arm cycling and arm swing activity (Zehr and Haridas 2003). Therefore, a special neural coupling occurs between the arms and legs when the arms move in alternation, which could have implications for gait rehabilitation.

Overall, the combined results comparing bilateral responses support a measurable neural coupling between the limb pairs and suggest that the strength of the neural coupling between the legs is relatively greater than for the arms (Dietz 2002; Zehr and Duysens 2004). This has been, in part, attributed to the crucial coupling between the legs for successful terrestrial locomotion and the specialized use of the arms for skilled hand movements. While this evidence supports a general active role of the arms during locomotor activities, specific details of arm to leg coupling have been gleaned using interlimb reflex studies.

## Mechanisms influencing arm and leg interactions during locomotion

### Interlimb effects may be uniquely specified to motor unit size

During rhythmic movement of all four limbs, the influence of the arms on reflex expression in the legs is superimposed on the dominant effect of the legs (Balter and Zehr 2007). This is based upon studies using cutaneous and H-reflex modulation as indices of neuronal activity related to locomotion, and early H-reflex studies were restricted to one phase of movement and to only a fixed H-reflex amplitude, and it was suspected that with all four limbs actively engaged in locomotion, contributions from the arms could be underestimated by somatosensory feedback from movement of the test limb.

Later studies used modulation of H-reflex amplitudes across the entire ascending portion of the M-H recruitment curve as a neural probe for interlimb coupling to separately evaluate the influences of rhythmic activity of the arms and leg on neuronal excitability of a stationary “test leg” (Mezzarane et al. 2011). This three-limb “reduced” locomotion approach revealed a large and significant influence of rhythmic arms and legs activity on H-reflex amplitudes at end power (e.g. mid-swing) and start of recovery phases (e.g. beginning of stance). However, the parameters (and thus motor unit populations) were differentially affected at each phase and task. For instance, a significant contribution of arm movement was detected for H_max_ (comprised of the largest of the reflexively activated motor units) at end power phase, but no changes were observed for other parameters that reflect the smaller, lower-threshold motor units.

Rhythmic arm activity is thus differentially manifested across motor unit populations for each phase of movement. These findings provide definitive evidence for interlimb coupling between cervical and lumbar oscillators in gating the excitability of reflex pathways to a leg muscle for different populations of lumbar motoneurons in humans. These results are complimentary to work from the cat, suggesting that descending locomotor drive may be differentially specified for different motor unit populations in the feline hindlimb (Tansey and Botterman 1996).

### Presynaptic inhibition is a major mechanism influencing spinal reflex excitability during interlimb locomotor activity

Presynaptic inhibition of transmission between Ia afferent terminals and alpha motoneurons (Ia PSI) is a major control mechanism associated with soleus H-reflex modulation during human locomotion (Capaday and Stein 1986; Crenna and Frigo 1987; Frigon et al. 2004). Rhythmic arm cycling modulates soleus H-reflex amplitude when the legs are stationary by increasing segmental Ia PSI (Frigon et al. 2004). In a reciprocal organization, leg cycling also modulates H-reflexes in stationary arm muscles (Zehr et al. 2007b).

Nakajima et al. (2013b) investigated whether Ia PSI contributed to the ascending conditioning effects from leg to arm. During rhythmic leg cycling, H-reflexes in forearm flexor muscles were suppressed by segmental Ia PSI (radial nerve; C-T interval = 20 ms) or facilitated (superficial radial (SR) nerve; C-T interval = 37–47 ms). Leg cycling suppressed forearm H-reflex amplitudes with the amount increased with radial nerve conditioning. In contrast, SR conditioning removed the suppression of H-reflex amplitudes induced by leg cycling. Both effects were observed with subthreshold conditioning stimulation that influenced H-reflex amplitudes but did not have significant post-synaptic effects. These related observations support conservation of neural control mechanisms underlying arm and leg coupling during locomotor behaviours in humans (Nakajima et al. 2011).

### Arm cycling alters excitability of reciprocal inhibition between ankle flexors and extensors

The effect of rhythmic arm movement on lumbar spinal cord excitability extends also to reciprocal inhibition. We investigated the effect of rhythmic arm cycling on modulation of short latency (~55 ms post-stimulus) reciprocal inhibition between ankle flexor (tibialis anterior, TA) and extensor (soleus, SOL) muscles (Dragert and Zehr 2013). Arm cycling significantly increased reciprocal inhibition in SOL but was unaltered in TA muscle.

Therefore, while descending signals arising from rhythmic arm movement significantly alter transmission in segmental reciprocal inhibitory pathways between ankle flexor and extensor muscles, it is differentially specified. This may be due to stronger descending supraspinal regulation to ankle flexors as opposed to extensor muscles during locomotion (Capaday et al. 1999). This could also be related to functional requirements during locomotion that have been observed with the differential effect of arm cycling on TA H-reflexes (Dragert and Zehr 2009).

### Roles for afferent feedback

The mechanics of a movement are closely linked to both neural output and afferent feedback. As described for the legs, the afferent control signals related to task mechanics are powerful modulators of the locomotor pattern and can reset the timing as well as shape the response. As such, it has been demonstrated that rhythmic arm movement can shape lower limb muscle activity (Hiraoka and Iwata 2006; Kawashima et al. 2008). Kawashima et al. (2008) observed that rhythmic arm swing has a powerful effect on soleus muscle activity during combined arm and leg movement.

Results from rhythmic arm cycling studies have left uncertainty about the role of afferent feedback from the arms and altered arm mechanics in modulating lower limb activity. Therefore, changes in afferent feedback in the arms may not have a sufficient modulatory effect on reflex amplitudes in stationary legs (Loadman and Zehr 2007; de Ruiter et al. 2010). It remains possible that this information is only incorporated when it could be functionally useful for postural or locomotor interaction while during static sitting there is little threat to lose balance. Alternatively, it may be possible that a difference in task constraints results in differential gating of afferent feedback from the arm (Hiraoka and Iwata 2006). That is, afferent signals related to the specific arm movement task such as from muscle, cutaneous or joint afferents could be crucial signals that modify leg muscle activity.

Alterations in excitability of the lumbar spinal cord induced by rhythmic activity in the arms may be relatively unaffected by afferent feedback. By varying arm cycling load and assessing the influence of vibration of arm muscles, the contribution of afferent feedback related to arm movement on the excitability of soleus H-reflexes was assessed (Hundza et al. 2012). There were no significant differences in the level of H-reflex conditioning across loading conditions or between conditions with and without arm muscle vibration.

Differences in descending conditioning during internally driven (“Active”) and externally driven (“Passive”) arm cycling were also studied (Hundza et al. 2012). In contrast to the clear conditioning effect seen during active cycling, involuntary passive arm cycling did not significantly modulate soleus H-reflex amplitude. These results suggest that central motor commands (supraspinal or spinal in origin) associated with frequency of arm cycling are the relatively dominant source of descending regulation (Hundza and Zehr 2009).

### Strength of interlimb connectivity depends on how many limbs participate

Spatial facilitation was used to examine possible convergence in common reflex pathways during rhythmic human locomotor limb movements (Nakajima et al. 2014). Cutaneous reflexes were evoked in TA muscle stimulating sural, distal tibial, or combined simultaneous stimulation of both nerves in random order. Reflexes were evoked during rhythmic stepping and arm swinging movement with both arms and the leg contralateral to stimulation (ARM&LEG), with just arm movement (ARM) and with just contralateral leg movement (LEG).

During ARM&LEG movement, early latency cutaneous reflex amplitudes by combined stimulation were significantly larger than simple mathematical summation found with sural or tibial alone. This extra facilitation seen during combined nerve stimulation was significantly reduced when performing ARM or LEG compared to ARM&LEG leading to the conclusion that locomotor rhythmic limb movement induces excitation of common interneuronal reflex pathways from cutaneous afferents innervating different foot regions.

This activity was most facilitated during ARM&LEG movement, suggesting a weighting according to the number of limbs directly engaged in human locomotor activity. This underscores both the importance of arm swing to support neuronal excitability in leg muscles and also has explanatory power for many of the nonlinear interactions observed during locomotion. Shared convergence in interlimb and segmental networks was studied using simultaneous cutaneous stimulation of the hand (superficial radial, SR, nerve) and foot (superficial peroneal, SP, nerve) during arm and leg cycling (Nakajima et al. 2013a).

**Fig. 4:**
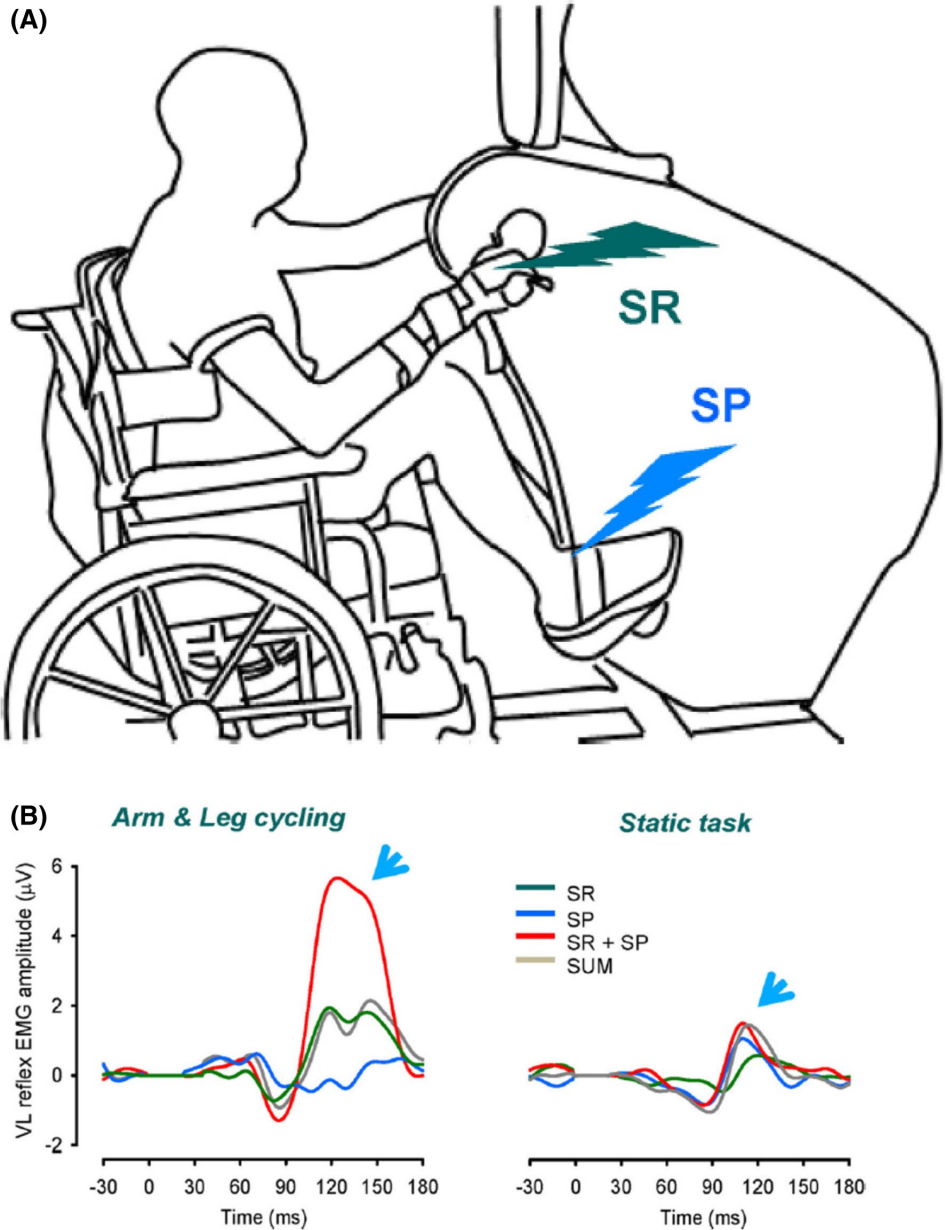
**a** Apparatus setup for arm and leg cycling with cutaneous nerve stimulation. Hand and foot stimulations are indicated by *lightning bolts*. b Reflexes evoked by simultaneous SR + SP stimulation arm and leg cycling show nonlinear amplification (see *arrow at left*) which is absent during static contraction (see *arrow at right*). *SR* superficial radial nerve, *SP* superficial peroneal nerve, *SR* *+* *SP* simultaneous combined stimulation of SR and SP reflexes. Adapted from Nakajima et al. (2013a)

Spatial facilitation of responses in the knee extensor vastus lateralis (VL) revealed that amplitudes of facilitatory responses with SR + SP stimulation were significantly larger than those for SP or SR stimulation alone and were also in excess of the simple mathematical summation of amplitudes from SP and SR trials (see Fig. 4). This extra facilitation was absent during static muscle activation and could not be accounted for by serial neuronal processing. Instead, this result suggested that simultaneous rhythmic activation of the arms and legs during ARM&LEG cycling activates shared interneurons in convergent reflex pathways from cutaneous inputs innervating the foot and hand.

Taken together, these studies highlight common interneuronal reflex pathways, which lead to excitability differences when the arms and legs work together during a locomotor task. This enhanced activity has functional implications for corrective responses during intact human locomotion and for translation to neurorehabilitation.

## Neuromechanical function of arm and leg interactions

Based on observations in human and non-human primates, Elftman (Elftman 1939, 1944) first proposed that arm swing during walking was necessary to counteract pelvic rotation. In a comprehensive kinetic analysis of walking with and without arm swing, Li et al. (2001) observed very little effect of suppressing arm swing on vertical or sagittal contact forces. Further, they suggested that the influence of arm swing on the kinetics of gait must be the effects on transverse forces and/or free vertical moments.

The stance foot produces a vertical moment that is largest just before heel strike of the opposite foot. When arm swing is restricted, the free vertical moment produced is much larger, presumably to compensate for the loss of the arm swing moment to counterbalance the lower limb swing. In a similar experiment, Umberger observed only small differences between kinematics and kinetics when walking with or without arm swing, with the main exception of larger differences in free vertical moments. Additionally, walking with arm swing was more energy efficient than walking without arm swing (Umberger 2008).

The function of neural connections between the arms and legs in humans may support the neuromechanical linkages. Segmental cutaneous reflexes in the legs are precisely gated and likely have a functional role in maintaining and restoring stability during walking (Zehr and Stein 1999; Haridas et al. 2005). During bipedal locomotion, arm movement is relatively unconstrained, and as the arms are not directly interacting with the ground, they are not in a mechanical position to immediately and directly modify stability. Haridas et al. (2005) reported a general facilitation of interlimb reflexes in the arms when the arms were crossed in front of the body during an unstable walking task. Facilitation appeared in several muscles, and a general role for interlimb reflexes in corrective responses during locomotion was postulated.

**Fig. 5:**
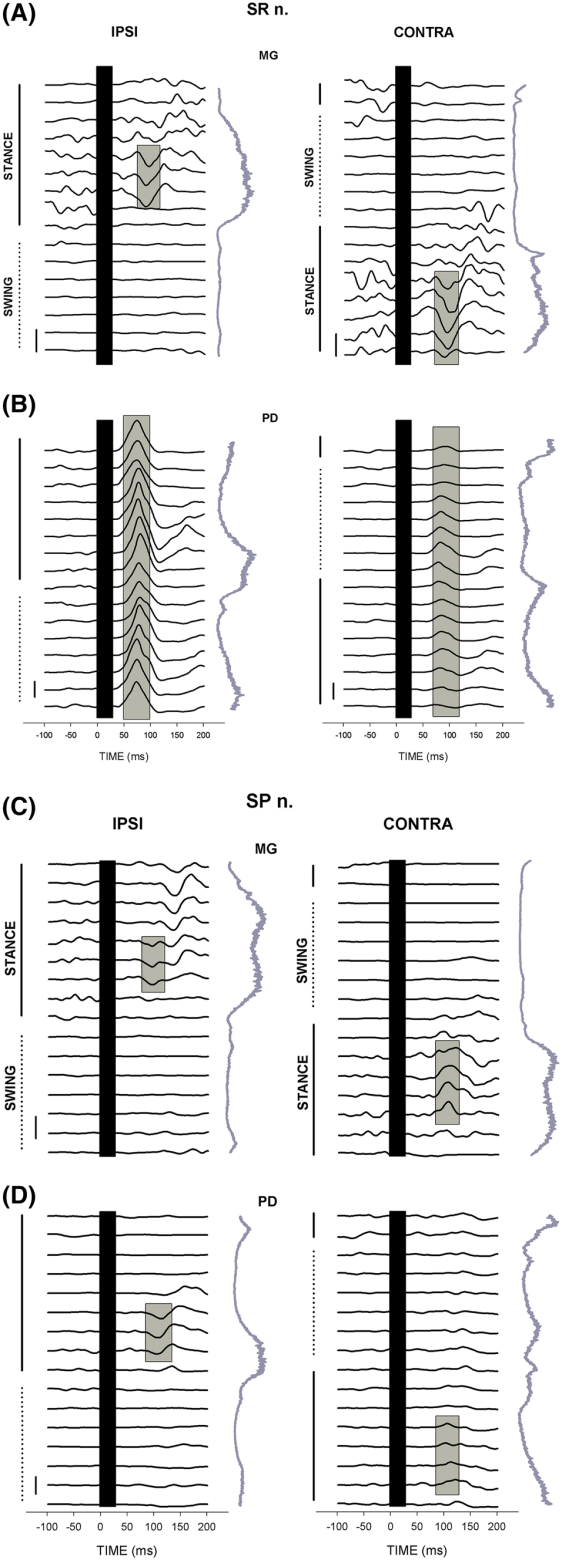
Reflex traces across 16 phases of walking in ankle plantarflexor muscle medial gastrocnemius (MG) and shoulder extensor posterior deltoid (PD) after stimulation at the wrist (SR nerve; **a**, **b**) and ankle (SP; **c**, **d**). Step cycle marking as shown and the vertical traces are background EMG envelopes for each muscle. Adapted from Haridas and Zehr (2003) J Neurophysiology

Interlimb reflexes can produce functional neuromechanical effects during locomotion in quadrupeds and humans. Cutaneous reflexes evoked in leg muscles by stimulating nerves of the foot and hand produce mechanical effects that are associated with functionally relevant changes in limb trajectory (Zehr and Duysens 2004). For example, cutaneous stimulation at the wrist resulted in ankle dorsiflexion at the stance to swing transition that could be functionally relevant to slow forward progression and minimize impact with an obstacle encountered by the outstretched arm (Haridas and Zehr 2003) (see Fig. 5).

Balter and Zehr found that rhythmic arm movement made a significant contribution to reflex expression in the legs during the power phase (comparable to heel strike in walking) or arm and leg cycling. They suggested that the contribution from the arm at this point could be explained by a possible reliance on multi-sensory integration to help guide the swing limb and ensure proper placement of the foot (Balter and Zehr 2007). This corresponds to similar effects from forelimb to hindlimb in the cat (Miller et al. 1975; Miller and Van Der Meche 1975).

Overall, studies on “reduced” locomotor preparations, such as arm and leg stepping or cycling, have shown that arm movement contributes to modulation of reflexes in the legs in a functional manner. Albeit, there is still a necessity for more robust mechanical analysis of responses occurring during these tasks. An important consideration for the interpretation of interlimb reflex function is to consider the behavioural context in which the reflex occurs. For example, Lamont and Zehr (2007) observed enhanced and task-specific cutaneous reflex responses during level walking, incline walking and stair climbing when the arms were in contact with an earth-referenced handrail. This suggests that interlimb reflexes in arm muscles may be enhanced in an environment where earth-referenced support is available and the arms can act directly to mechanically stabilize the body (Lamont and Zehr 2006, 2007).

Behaviourally, the arms and legs are coordinated during locomotor activities like walking, creeping and swimming. Wannier et al. (2001) showed that during all of these activities, an integer frequency relationship was maintained between the limbs, suggesting a coupling between neuronal circuits controlling arm and leg movements that corresponds to that observed in systems consisting of two coupled oscillators (von Holst 1954).

Neuronal connections between the cervical and lumbar pattern generators are retained in bipedal humans, despite the fact that the arms do not directly generate propulsion during walking. It is likely that these connections are functional in coordinating whole-body responses to perturbations during gait; however, the specific role of these intergirdle pathways has yet to be determined.

**Fig. 6:**
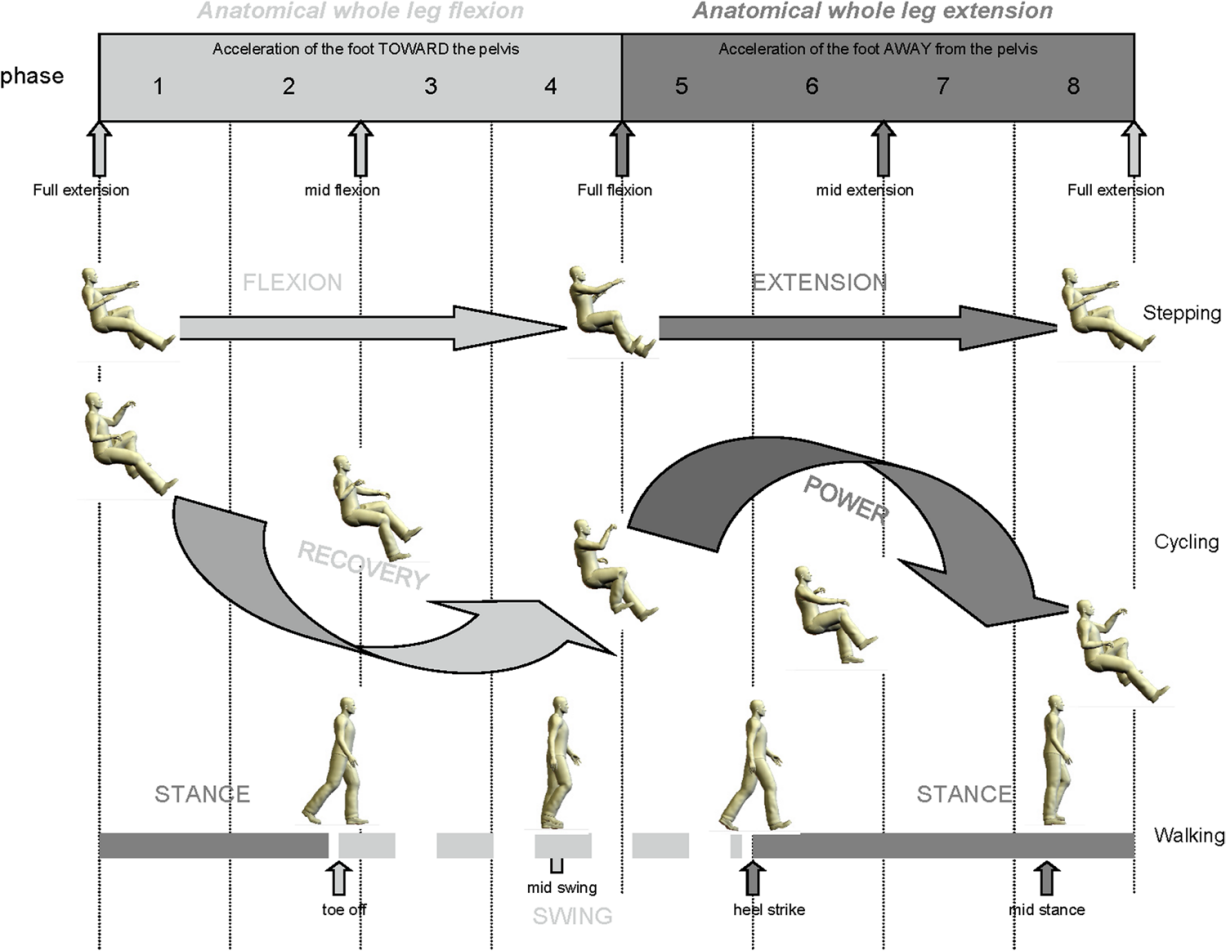
Relative functional phases of gait for recumbent stepping, cycling and walking. Flexion–extension for stepping, recovery–powder for cycling, and stance–swing for walking refer to the ipsilateral (*right*) leg and correspond to the images in the figure. The topmost terms of biomechanical flexion and extension refer to the overall motion of the limb towards or away from the body, respectively. Adapted from Zehr et al. (2007c) J Physiol

It has been suggested that making use of cervicolumbar connections could be used in rehabilitation following neurotrauma (Dietz 2002; Ferris et al. 2006; Zehr et al. 2009). For example, by incorporating integrated arm and leg training in gait recovery protocols, such protocols could use adjunct activities such as arm and leg stepping (Rebecca et al. 2007; Zehr et al. 2007a; Nakajima et al. 2014), arm and leg cycling (Andersen et al. 2014, 2007a; Balter and Zehr 2007; Sasada et al. 2010; Nakajima et al. 2013b) or exaggerated arm swing during exoskeleton stepping (La Scaleia et al. 2014). The analogous relationships to phases of the locomotor cycle for arm and leg stepping, cycling, and walking have been previously established, to better allow for translation to locomotor rehabilitation (see Fig. 6).

## Translation to human locomotor rehabilitation

“You might wanna think about…maybe, eh…moving your arms a little when you walk…You know, sort of swing them, so you’re not lurching around like a caveman.”

–Conversation between Elaine and Co-worker from NBC TV “Seinfeld” episode “The Summer of George” (Ackerman 1997)

Given the similarities between the organization of the motor systems and behaviours between non-human primates and humans (Falgairolle et al. 2006; de Seze et al. 2008), it has been proposed that non-human primates may provide a unique model for evaluating efficacy and safety of treatments for humans after neurotrauma, thus hastening the translation of interventions. In fact, it was demonstrated that the primate has the ability to rapidly regain locomotor performance and to a lesser degree fine foot motor skills, following a reduction in supraspinal control (Courtine et al. 2005, 2007).

As outlined above, the available evidence suggests that humans, like other animals, possess pattern generating networks within the spinal cord that are capable of coordinating the basic walking pattern. However, a few words of caution are necessary before concluding that the neural control of walking is completely similar between humans and quadrupeds. There is a general and reasonable consensus that adult human locomotion is under more supraspinal control than in other animals (reviewed in (Capaday 2002; Nielsen 2003; Yang and Gorassini 2006). This has been poignantly demonstrated by studies, showing that body-weight support treadmill training is far more effective in retraining gait in quadrupeds (Lovely et al. 1986; Barbeau and Rossignol 1987), as compared to bipedal humans (Dietz et al. 1994a; Dobkin et al. 1995; Harkema et al. 1997; Mehrholz et al. 2008; Moseley et al. 2008) or non-human primates (Eidelberg et al. 1981; Fedirchuk et al. 1998). Often improvements in the walking of individuals after stroke or incomplete spinal cord injury can be partially correlated with increased corticospinal drive to muscles and/or increased activity in cortical areas (Dobkin 2004; Dobkin et al. 2004; Winchester et al. 2005).

The studies above raise significant implications for recovery of walking after neurotrauma because the recovery of arm muscle coordination during rhythmic movement could assist with recovery of leg muscle activity. Behrman and Harkema (Behrman and Harkema 2000) evaluated case studies of locomotor retraining after spinal cord injury and suggested that using the arms for postural and weight-bearing activity (e.g. on parallel bars or handrails), as is commonly applied in therapy, may inhibit rhythmic stepping with the leg. In contrast, a normal reciprocating arm swing such as found in natural walking may facilitate stepping. Therefore, they suggest that arm swing is an important component needed to help improve motor output for the legs during walking. These observations support incorporating rhythmic arm movement paradigms for locomotor rehabilitation after neurotraumatic injury in humans. Ferris et al. (2006) previously argued that in order to harness interlimb neural coupling, gait rehabilitation therapy should incorporate simultaneous arm and leg rhythmic activity after neurotrauma.

An important consideration in applying locomotor retraining in rehabilitation is the extent to which the training task transfers to the intended functional task. As a rehabilitation strategy tested in the mouse SCI model, Smith et al. (2006b) examined the potential enhancement of functional recovery of all four limbs using swim training with and without cutaneous feedback (centrifuge tubes floating in the water that did not provide support). Cutaneous feedback enhanced the improvement of swimming ability but did not affect the improvement of walking capabilities. In a further study, Magnuson et al. (2009) examined the kinematics of rats during swim training following SCI and found that the velocities of hindlimb movements remain significantly below normal. These experiments illustrated that, although swim training resulted in improved swimming performance and increased hindlimb activity, it did not transfer significantly to over-ground stepping (Smith et al. 2006a, b; Magnuson et al. 2009).

A limitation to transfer effects of swim training to stepping may have been the lack of body-weight support training with coincident afferent feedback that may be essential for terrestrial locomotor retraining but are reduced in swimming. To take advantage of voluntary and quadrupedal stepping in an environment which provided dynamic body-weight support (buoyancy) along with substantial limb-loading and cutaneous feedback, Kuerzi et al. (2010) employed a model of step training in shallow water. Shallow-water stepping abilities were enhanced, but again with little transfer to over-ground stepping, again highlighting the need to consider features of task specificity in retraining following neurotrauma.

Related work clearly shows the negative training effect of reduced mobility induced by hindlimb immobilization in a specially constructed wheelchair on the recovery of locomotor function in the rodent. Caudle et al. (2011) found that such wheelchair rats showed a significant decline in locomotor function following SCI compared to mobile controls. They suggested that immobilization might hinder or conceal the normal course of functional recovery after neurotrauma, an effect that could not be overcome by the activity of the forelimbs during recovery.

The above underscores the importance that neural commands related to the production of rhythmic arm movement could play in assisting with accessing the neural circuitry coupling the arms and legs during locomotor retraining in humans. A key caveat, however, is the extent to which pathways mediating arm and leg interactions remain accessible after neurological damage like spinal cord injury and stroke.

### Interlimb neural coupling after stroke

In rhythmic arm cycling, motor output and modulation patterns of cutaneous reflexes evoked in both the more affected and less affected arms were studied in chronic stroke. Partial preservation of essential rhythmic patterning of arm muscle activation and neural control of spinal cord excitability appears to persist in chronic stroke but shows a somewhat “blunted” effect (Zehr et al. 2012). The overall implication is that the putative spinal contributions to rhythmic human arm movement remain accessible, which has translational implications for post-stroke rehabilitation. This corroborated the effects found earlier for descending modulation of spinal cord excitability. Interestingly, larger deficits are seen after stroke in discrete arm reaching actions than in rhythmic locomotor actions, suggesting a subcortical regulation of the latter (Leconte et al. 2016).

Previously, Barzi and Zehr (2008) showed that H-reflex amplitudes in the MA SOL after stroke could be modulated by arm cycling. The effect was weaker than in NI control subjects but did demonstrate maintained signalling of rhythmic arm movement to the lumber spinal cord in those with chronic stroke. This led to a follow-up experiment assessing the contribution of presynaptic inhibition to interlimb regulation using stretch reflexes in chronic stroke participants (Mezzarane et al. 2014). Interestingly, we observed bidirectional reflex modulation induced by arm cycling that produced facilitation in some and suppression in other participants after stroke.

The relative conditioning effect of arm cycling on stretch reflex amplitude did not differ between the more affected and less affected legs. Since there may be a differential effect of arm cycling on stretch and H-reflexes due to Ia presynaptic inhibition (Palomino et al. 2011), alterations in stretch reflex amplitude modulation after stroke may reflect changes in both presynaptic afferent transmission mechanisms and fusimotor control.

**Fig. 7:**
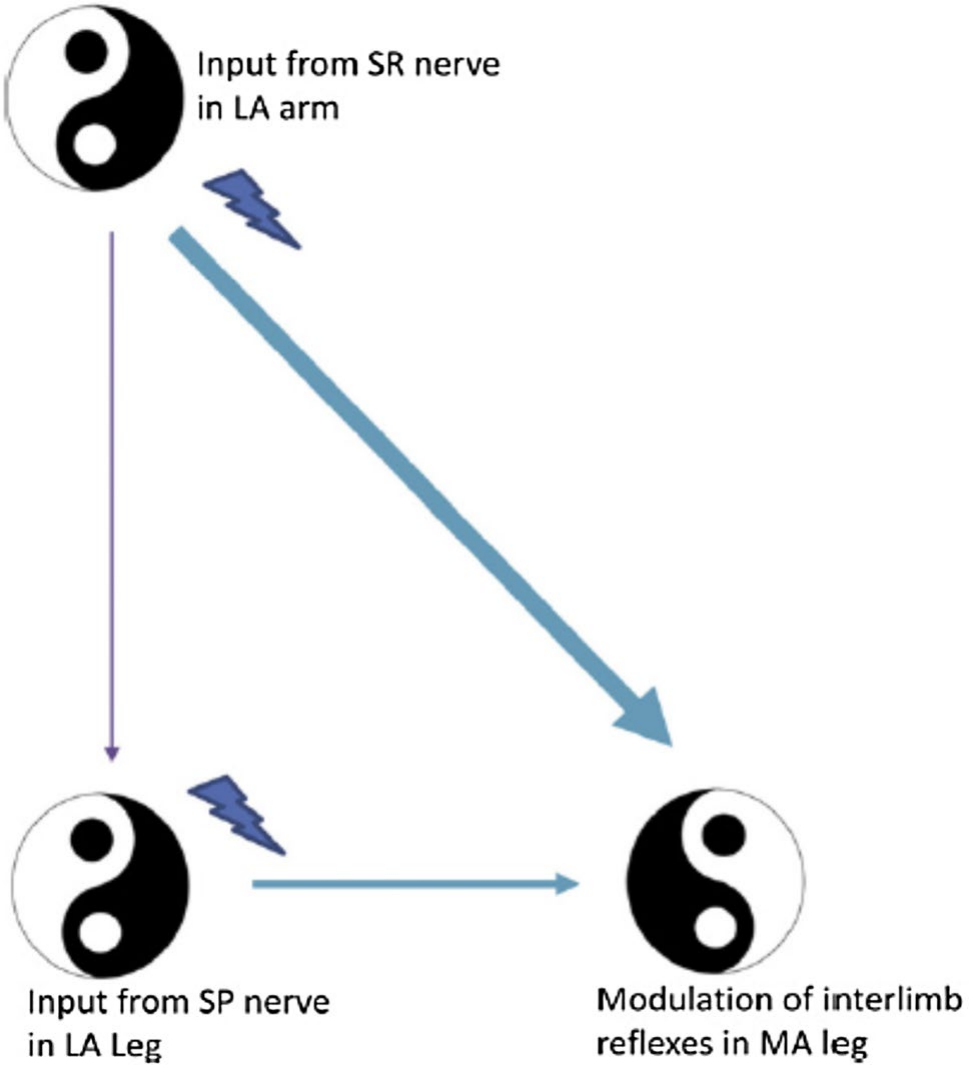
Schematic representation of strength of interlimb cutaneous reflexes after stroke. Remote sensory input modulates output of the more affected leg. Adapted from Zehr and Loadman (2012) Clin Neurophysiol

It is important to note that some form of somatosensory interlimb coupling persists after stroke (Zehr and Loadman 2012). It was recently shown that interlimb cutaneous inputs may access coordinated reflex pathways in the more affected arm or leg during walking after stroke. Importantly, activation in these pathways could elicit activation in ankle muscles that might counter foot drop during the swing phase of walking (see Fig. 7). Although a portion of interlimb coupling appears to be maintained after stroke, it is also important to assess whether coupling persists across distinct but functionally similar locomotor tasks.

It is generally understood that the basic pattern of arm and leg movements during rhythmic locomotor tasks is supported by common central neural control from spinal and supraspinal centres in neurologically intact participants. However, it was unclear to what extent this is maintained following a cerebrovascular accident (Klarner et al. 2015). Recently, Klarner et al. (2015) found shared systems from interlimb cutaneous networks facilitating arm and leg coordination that persisted across locomotor tasks using all four limbs: arm and leg cycling using a stationary ergometer and walking on a motorized treadmill.

Background EMG was similar between walking and arm and leg cycling, especially in the distal leg muscles. Cutaneous reflexes were evident and modified in a similar modulation pattern between locomotor tasks in both the less and more affected limbs, suggesting activity in related control networks between tasks. After a stroke, common neural patterning from conserved subcortical regulation supports the notion of a common core in locomotor tasks involving arm and leg movement (Klarner et al. 2015). This has translational implications for rehabilitation where concurrent arm and leg cycling might be usefully applied to improve walking function.

An important consideration in translation is the potential role for exaggerated arm motion to affect locomotor rehabilitation. This issue may be increasingly important following neutrotraumatic injury since walking speeds are typically reduced. It is known that, during low-frequency stepping of neurologically intact individuals, arm swing is altered compared to high-frequency steps (Webb et al. 1994). (MacLellan et al. 2013a). Although individuals vary based on the speed, arm swing changes from reciprocal unilateral swing in unison with the contralateral leg at high frequencies, to bilaterally coupled arm swing during low-frequency stepping. This altered pattern of arm swing during stepping at a slower pace does not require coordination of the arms to facilitate stepping of the legs and therefore revert to a natural pendulum frequency. However, it is possible that during low-speed walking exhibited following neurotrauma, the capacity of arm swing to facilitate stepping may be increased, although this remains to be substantiated. Detailed work has also been done in human crawling and examining arm and leg coupling across different frequencies (MacLellan et al. 2013a). Related prior work investigating interlimb coupling effects during arm cycling suggested a minimum arm frequency of ~0.8 Hz for optimal engagement of cervicolumbar interactions (Hundza and Zehr 2009).

Application of the approaches described above has recently been assessed in a post-stroke intervention using arm and leg cycling to test its efficacy as an agent for inducing targeted neural plasticity (Klarner et al. 2016a) and improving neurological integrity and locomotor ability (Klarner et al. 2016b). Chronic stroke (greater than 6 months post-lesion) participants performed 30 min of arm and leg cycling training (at a frequency of ~0.9 Hz) three times a week for 5 weeks. Changes in walking function were assessed with: (1) clinical tests; (2) physical performance (e.g. joint range of motion and muscle activity patterns) and strength; and (3) neurophysiological integrity measures (cutaneous and stretch reflex excitability). A multiple baseline (3 pretests) within-subject control design was used (Klarner et al. 2014). The results revealed that arm and leg cycling improved neurological function and walking ability, inferred by changes in walking characteristics, reflex function, ankle strength, and increased distance walked in the 6-min walk test. These results suggest that arm and leg cycling training, an accessible and cost-effective training modality, could be used to improve interlimb coupling and walking ability after stroke (Klarner et al. 2016b). This approach could also have application in other disorders where interlimb coupling is disrupted, such as Parkinson’s disease, cerebral palsy and multiple sclerosis. Parkinson’s disease is particularly relevant given the documentation on disrupted arm and leg swing coordination (Huang et al. 2012; Kwon et al. 2014; Lewek et al. 2010) and potential prodromal deterioration of arm swing.

## Summary and conclusion

**Fig. 8:**
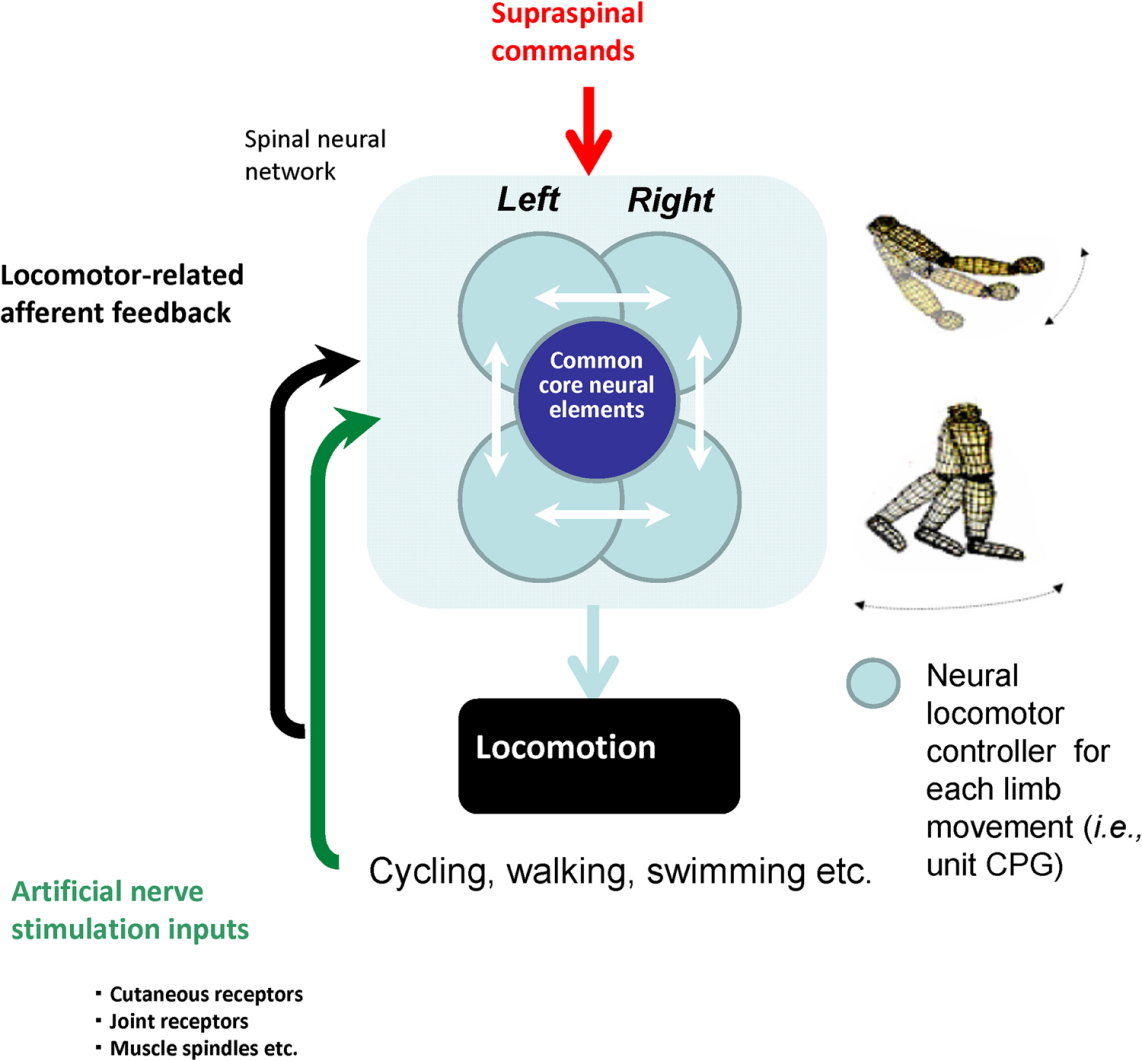
Cartoon schematic indicating “reconstruction” of walking ability after neurotrauma. Idealized interrelationships between supraspinal regulation, spinal patterning and reflex control, and afferent feedback are as indicated

Evidence accumulated in humans suggests that the basic neural elements controlling and coupling the arms and legs during coordinated rhythmic movements are similar to those in habitually quadrupedal animals. It is possible that these interlimb connections may be exploited to facilitate locomotor rehabilitation after neurotrauma. Framed under the concept of “reconstructing” locomotor ability, this is shown schematically in Fig. 8.

Of specific interest to walking retraining is the effect of arm activity on leg neural circuitry. That is, can movement of the arms be used to assist in lower limb locomotor retraining following stroke or spinal cord injury? There is exciting evidence supporting an active role of the arms in combined arm and leg rhythmic movement.

Work continues to refine our understanding of the neural control of locomotion and mechanisms of interlimb coupling before this information can be properly implemented in rehabilitation. This is, in part, due to the fact that coordinated rhythmic movement of all four limbs, like walking, running and cycling, is not simple movement. These tasks require specific neural control signals that precisely activate muscles to produce an appropriate multi-limb coordinated biomechanical output. Further, sensory feedback of body movements and physical interactions may result in powerful signals that alter control parameters. There is a fundamental, bidirectional coupling between the neural control and the mechanics of rhythmic movement that is difficult to separate.

In closing, more than 100 years later, the words by Sir Charles Sherrington that “all parts of the nervous system are connected together” [found in his 1906 classic “The Integrative Action of The Nervous System” (Sherrington 1906)] resonate here. Determining the necessary and sufficient conditions to enhance interlimb coordination during locomotor rehabilitation requires an appreciation of the sublime interactions between neural control, mechanical action and behavioural context.
